# The burden of anthropometric failure and child mortality in India

**DOI:** 10.1038/s41598-020-76884-8

**Published:** 2020-12-02

**Authors:** Junaid Khan, Sumit Kumar Das

**Affiliations:** 1 International Institute for Population Sciences, Govandi Station Road, Mumbai, Maharashtra 400088 India; 2 Department of Biostatistics, National Institute of Mental Health and Neurosciences, Bengaluru, 29 India

**Keywords:** Medical research, Risk factors, Socioeconomic scenarios, Nutrition

## Abstract

The public health burden of nutritional deficiency and child mortality is the major challenge India is facing upfront. In this context, using National Family Health Survey, 2015–16 data, this study estimated rate of composite index of anthropometric failure (CIAF) among Indian children by their population characteristics, across states and examined the multilevel contextual determinants. We further investigated district level burden of infant and child mortality in terms of multiple anthropometric failure prevalence across India. The multilevel analysis confirms a significant state, district and PSU level variation in the prevalence of anthropometric failures. Factors like- place of residence, household’s economic wellbeing, mother’s educational attainment, age, immunization status and drinking water significantly determine the different forms of multiple anthropometric failures. Wealth status of the household and mother’s educational status show a clear gradient in terms of the estimated odds ratios. The district level estimation of infant and child mortality demonstrates that districts with higher burden of multiple anthropometric failures show elevated risk of infant and child mortality. Unlike previous studies, this study does not use the conventional indices, instead considered the CIAF to identify the exact and severe form of undernutrition among Indian children and the associated nexus with infant and child mortality at the district level.

## Introduction

Nutritional deficiency among the children is one of the major public health concerns in the developing countries and India is not an exception to this problem. Nutritional status of a child under age five are commonly measured in terms of three different standard anthropometric measures based upon height, weight, sex and age of the children. These three anthropometric measures are stunting, underweight and wasting. As per the standards of World Health Organization (WHO), nutritional status of the children is determined in terms of the age-sex standardized anthropometric z-scores namely, height for age (HAZ), weight for age (WAZ) and weight for height (WHZ) z-score. A child with less than -2SD (standard deviation) of the median value of the HAZ score of the reference population is considered as stunted. Similarly, underweight is defined as less than -2SD of the median value of the WAZ score and considering the same cut-off of the WHZ score, wasting is defined^1^. All the three anthropometric z scores are compared against an international reference population and these three measures give three different patterns of nutritional deficiency among the children^1^. These measures are indicative of child’s poor nutritional status due to inadequate dietary intake and termed as the measures of undernutrition and does not necessarily inform about the malnutrition status of the child^2^. As we know, malnutrition as a measure defines all the types of optimal nutritional status including energy undernutrition and over-nutrition^2^. Parallel to the merits of these scientific measures of stunting, underweight and wasting, one certain demerit to these measures are that the measures may overlap and a particular child may fall in two groups or even in all the three groups simultaneously. In the year 2000, Peter Svedberg gave a solution to this problem building a model to classify the type of undernutrition and to estimate the exact prevalence of child undernutrition^3, 4^.

Though nutritional deficiency may occur in any stage of life but in a particular population, children are more vulnerable and susceptible than the adults to be malnourished due to various reasons like- inadequate dietary intake, infection to diseases, lack of appropriate health care, and inequitable distribution of food within the household^5^. Globally, malnourishment is the single largest factor to contribute to the burden of child mortality and is associated with half of the child deaths^6, 7^. Recent estimates on child undernutrition shows that, 156 million children are stunted, 93 million are underweight and 50 million are wasted under five years of age^8^. And struggling with the undernutrition situation, India accounted 62 million stunted children in the year 2016 (40 percent of the global share)^9^.

India carries a significant burden of undernourished children and there exists a large spatial heterogeneity in terms of child stunting, underweight, wasting and anemia prevalence across districts of India^10, 11^. Of the total children in India, 38 percent are stunted, 36 percent are underweight and 21 percent are wasted indicating the present alarming situation of undernutrition among the Indian children^12^. To combat with the situation, policies at national and at sub-national level are formulated to reduce the burden of child undernutrition and the associated morbidities among the children. Since the International Conference on Population and Development in 1994 at Cairo, the paradigm shift in India’s Population Policy embodied the National Population Policy in the year 2000 where child health and nutrition received much emphasis to be targeted. Government of India (GOI) took several initiatives to improve the living standard and socio-economic status of the population, to improve the maternal and child health with an emphasis to family planning and reproductive health services with a long term objective for sustainable economic growth, social development and environmental protection.

Child mortality is another demographic phenomenon which is of immense public health concern globally. Child mortality is measured by two important indicators-under five mortality (probability of dying before reaching age five) and infant mortality (probability of dying before reaching age one). Under five mortality of any given population is one of the important indicators which reflects the health and socio-economic status of that population group in any given period of time^13^. Globally, under five mortality rate declined from 150 per 1000 live birth in 1970 to 67 per 1000 live births in 2010^13^. Parallel to global reduction in under-five mortality, India has also shown a consistent decline in infant mortality and under five mortality but India failed to meet the target of reducing under-five mortality by two-third to achieve the Millennium Development Goal four (MDG-4) by 2015. Though India has shown a decline from 190 per live births in 1990 to 64 per 1000 live births in 2011^14^. Since the Alma Ata declaration in 1978, Government of India took several initiatives to reduce child mortality. The National Diarrhoeal Disease Control Programme began in 1978. The Universal Immunization Programme and oral rehydration therapy (ORT) started in 1985. The Acute Respiratory Infection (ARI) Control programme started in 1990.

Poor nutritional status among children is associated with death and solely contributes to half of all the deaths among the children in developing countries^15–17^. Previous studies also established the association between malnutrition and childhood morbidity suggesting that children with anthropometric failure are at higher risk of childhood morbidity^7, 18–20^. Thus this study examined the undernutrition prevalence in terms of anthropometric failures which gives the exact estimate of undernutrition in any given period of time in the sub-population and the toll of infant and child mortality across districts of India. Whilst, it is important to estimate the exact pattern of undernutrition among the children for a geographically diverse country like India, few studies and no population level studies have investigated the potential linkages between composite index of anthropometric failure (CIAF) unlike the regular measures of undernutrition like stunting, wasting and underweight with infant and under five mortality across the districts of India. While conducting the experiment, this study hypothesized that districts with higher prevalence of multiple anthropometric failures carry higher burden of infant and child mortality. Beforehand, we identified the potential risk factors of anthropometric failure and further examined the variations in child and infant mortality across the districts in terms of the district level burden of multiple anthropometric failures.

## Methods

### Data source

Unit level data from National Family Health Survey of 2015–16 (NFHS-4) has been used in this study. The data is publicly available at https://dhsprogram.com/data/ and thus requires no ethical approval further. International Institute for Population Sciences (IIPS), India being the nodal agency approved all the survey protocols. National Family Health Survey round four, 2015–16 is one of the largest demographic and health survey being carried out in 640 districts of India. . The sample size of the survey constitutes of 6,99,686 women and 1,03,525 men from 6,01,509 households across India. The survey is intended to provide important indicators on population, health and nutrition. Necessary information on socio-demographic characteristics, marriage, fertility, children’s immunizations and childcare, nutrition, contraception, fertility preference, sexual behaviour, attitudes towards gender roles, HIV/AIDS, anthropometric measurements are collected. NFHS-4 adopts two stage stratified probability proportional to size sampling design where census villages and urban blocks are the first stage unit for rural and urban areas respectively, and the households are the second stage unit^21^. A sample of 205,480 children constitutes the units of analysis of this study. Annexure-1 gives the flow chart showing the analytical sample.

## Unit level study variables

### Outcome variable

The key outcome variable of the unit level analysis is the composite index of anthropometric Failure (CIAF), which is calculated using stunting, wasting, underweight status of the study children. The CIAF variable is generated using the definition of anthropometric failure^4^. The classification is shown below-

**Table:** 

Group	Description	Wasting	Stunting	Underweight
A	No Failure	No	No	No
B	Wasting Only	Yes	No	No
C	Wasting and Underweight	Yes	No	Yes
D	Wasting, Stunting and Underweight	Yes	Yes	Yes
E	Stunting and Underweight	No	Yes	Yes
F	Stunting Only	No	Yes	No
Y	Underweight Only	No	No	Yes

Note. As per definition, the grouping of CIAF does not contain a group of children with both stunted and wasted.

### Exposure variables

For the unit level analysis to examine the determinants of different forms of anthropometric failures, following variables are included in the study as per the previous literatures: Place of residence (rural, urban), wealth index (poorest, poorer, middle, richer& richest), social groups (SC, ST, OBC, others), place of delivery (home, institutional), educational attainment of mother (no education, primary education, secondary education and higher education), birth order (first, second, third, fourth or higher), child age in years and sex of the child (male, female), immunization status of the child (no/partial, full), safe drinking water facility (improved, unimproved) and sanitation facility (improved, unimproved).

## District level study variables

### Outcome variables

Two main outcome variables of the district level analysis are infant mortality rate (IMR) and child mortality (CMR)^22^. These two measures are important indicators of child health and are widely used to document the progress in the achievement of the fourth Millennium Development Goal (MDG-4: a commitment to reduce under-five mortality by two-thirds, between 1990 and 2015). IMR measures the probability of death before the child’s first birthday whereas CMR measures the probability of death after first birthday and before the child’s fifth birthday. IMR and CMR is estimated using information on the births and deaths at district level from NFHS-4 dataset using the DHS method of estimating infant and child mortality rates. DHS method adopts the synthetic cohort probability method to estimate the mortality rates. The details of the methodology is discussed elsewhere^23^.

### Exposure variables

District level percentages of all the concerned variables are estimated and validated through NFHS-4 report. The set of independent variables included in the district level study are the following: proportion of rural children, proportion of poor-the lowest two wealth quintiles- of the already calculated wealth index using household assets information in the NFHS-4 dataset^24, 25^. Proportion of Scheduled Caste (SC) and Scheduled Tribe (ST) children, proportion of mother with no education, proportion of female child, proportion of home delivery, proportion of no/partial immunization, proportion of children with no access to safe drinking water and proportion of children with no access to improved sanitation.

## Statistical analyses

### Multilevel analysis

A four level hierarchical model is considered in this analysis because each of the level has a specific topographical, social and environmental importance that could potentially influence child’s nutritional status^26, 27^. Given a hierarchical structured data, multilevel modeling is always advantageous and helps to estimate the variance at different levels that are conceptualized under the study framework^27^ The sampling design of NFHS data has its own hierarchy where children are apparently nested within PSUs, PSUs are nested within districts and districts are nested into states. According to NFHS-4, PSUs are the villages in rural areas and census enumeration blocks in urban areas and hence it is likely that children within PSUs possess similar characteristics and differs between PSUs. As per the Indian federal structure, district is the second level administrative area and state is the first-level administrative area where health specific governmental policies and programs are implemented and interventions take place. So taking care of the survey design and the hierarchical nature of the data, we employed a four-level multilevel structured modeling with children at level 1, nested within PSUs at level-2, nested within districts at level-3 and finally districts, nested within states at level 4 in order to account for the cluster sampling design and decompose the variation in child nutrition at child, PSU, districts and at state level^28^. Here we employed four level random intercept binary logistic model to estimate the corresponding probability $({\uppi }_{{{\text{ijkl}}}} )$ for the i-th child from the j-th PSU, k-th district and l-th state who suffer from any specific forms of multiple anthropometric failure. And the corresponding probability could be denoted as ${\uppi }_{{{\text{ijkl}}}}$ = Pr(${\text{y}}_{{{\text{ijkl}}}} = 1)$ as: ${\text{logit}}\left( {{\uppi }_{{{\text{ijkl}}}} } \right) = {\upbeta }_{0} + \beta_{m} X_{ijkl} + \left( {{\text{f}}_{{0{\text{l}}}} + {\text{v}}_{{0{\text{kl}}}} + {\text{u}}_{{0{\text{jkl}}}} + e_{0ijkl} } \right)$ where m is the number of independent variables included in the model^29^. The parameter, ${\upbeta }_{0}$ , is the intercept which is the only fixed term in the model. And ${\text{f}}_{{0{\text{l}}}}$, ${\text{v}}_{{0{\text{kl}}}}$, $u_{0jkl}$ and $e_{0ijkl}$ are the random effects, the residual differentials at state, district, PSU and at child level respectively. Random effects are assumed to be independent and normally distributed with a mean of 0 and variance of ${\upsigma }_{{{\text{f}}0}}^{2}$ ${\upsigma }_{{{\text{v}}0}}^{2}$ , ${\upsigma }_{{{\text{u}}0}}^{2}$ and ${\upsigma }_{{{\text{e}}0}}^{2}$ respectively^29^. These variances quantify the between-states $({\upsigma }_{{{\text{f}}0}}^{2} )$, between-districts $({\upsigma }_{{{\text{v}}0}}^{2} ),$ between- PSUs $({\upsigma }_{{{\text{u}}0}}^{2} )$ and between-children (${\upsigma }_{{{\text{e}}0}}^{2} )$ variations respectively in the log odds of the events under study, conditioned on the characteristics at different levels.

As the variance estimate of the lowest level cannot be obtained from the model, rest of the variances for the next higher levels are assumed to be a function of the binomial distribution. From the estimated variance of the random effects, proportion of variation known as the variance partitioning coefficients (VPCs) are calculated^29, 30^. As child is the lowest level of this study framework, we assumed the between-children variation to be the variance of the standard logistic distribution as ${\uppi }^{2} /3 = 3.29$^30, 31^. Thus for any level *x*, the VPC can be calculated using the following formula: ${\text{VPC}}_{x} = {\upsigma }_{x0}^{2} /({\upsigma }_{{{\text{f}}0}}^{2} + {\upsigma }_{{{\text{v}}0}}^{2} + {\upsigma }_{{{\text{u}}0}}^{2} + 3.29)$.

First-order predictive (or penalized) quasi-likelihood (PQL) estimation is used to approximate the linearization with the help of a Taylor series expansion which transforms the discrete binary response model to continuous model^32^. MLwiN version 2.34 (Centre for Multilevel Modelling, University of Bristol, Bristol, UK)^33^ software is used to obtain all the estimates of multilevel modeling.

### Generalised linear model estimation of infant & child mortality

Usual linear regression assumes normal distribution of the distribution of error. Whereas, if the data is not normal, simple linear regression cannot give accurate result and an application of OLS may lead to unbiased estimation. To cope with the problem, a generalized linear model (GLM) is used which assumes that distribution of the study variable is a member of exponential family of distribution^34^. Every GLM model has three components, namely, random component, systematic component and the link function^35, 36^. The random component depicts the probability distribution of the response variable; systematic component is the linear combination of the set of predictor variables and lastly the link function which is actually a transformation of the expectation of the response variable specifies the link between the random and the systematic component. In this study, the response variables are assumed to follow a Poisson distribution. And a log link function is considered. The form of the generalized linear model used in this study is given as:

$$g\left( {\mu_{i} } \right) = \ln \left( {\mu_{i} } \right) = f\left( {X_{i} ,b} \right)$$

where $\mu_{i}$ is the random component in the model follows a Poisson distribution with parameter $\lambda_{i}$ . $f\left( {X_{i} ,b} \right)$ denotes the systematic component which is a linear combination of the independent variables . And the random and systematic components are linked with natural log. The model performances are evaluated using the Akaike Information Critria (AIC). We used Stata version 14.1 MP (StataCorp LP, College Station, TX, USA) to analyse the data.

## Results

A total of 205,480 children are included in this study (Table 1). Of the total children, 24% children are from rural areas. The religion composition of the study children shows that 72% children are Hindu, 16% children are Muslim and 12% children belong to other religion category. Half of the children belong to the two lowest wealth quintiles and only 14% of the surveyed children are from the richest wealth quintile. About 31% of the children’s mother does not have any formal education. The social class divide of the children shows that 20% of the children belong to Scheduled Caste (SC) category and 21% of the children belong to the Scheduled Tribe (ST) category. Of the total surveyed children, 36% are of first ordered births and 17% of them are of fourth or higher ordered births. Fifty two percent of the children are male. Place of delivery information of the children tells that almost one-fourth of the children were delivered in home without any skilled birth attendant. Of the total surveyed children, 18% are of age less than one year. Only 51% of the children aged 0–59 months are fully immunized. Around 13% of the children do not have access to safe drinking water and 50% of the children do not have access to improved toilet facility in their houses.

**Table 1:** Description of the study variables, India, 2015-16

Variables	Sample Size	Distribution	Variables	Sample size	Distribution
CIAF*			**Birth order**		
No failure	91,901	44.7	First	73,865	36.0
Wasting only	12,306	6.0	Second	63,748	31.0
Wasting & underweight	16,583	8.1	Third	33,647	16.4
Wasting, stunting & underweight	13,288	6.5	Fourth and more	34,220	16.7
Stunting & underweight	37,053	18.0	**Sex of the child**		
Stunting only	29,470	14.3	Male	1,06,385	51.8
Underweight only	4,879	2.4	Female	99,095	48.2
**Place of residence**			**Place of delivery**		
Rural	49,023	23.9	Home	50,808	24.7
Urban	1,56,457	76.1	Institutional	1,54,672	75.3
**Wealth quintile**			**Child’s age**		
Poorest	54,325	26.4	Less than 1 year	36,990	18.0
Poor	47,990	23.4	Between 1 and 2	41,147	20.0
Middle	40,982	19.9	Between 2 and 3	41,288	20.1
Richer	34,279	16.7	Between 3 and 4	43,795	21.3
Richest	27,904	13.6	Less than 5	42,260	20.6
**Mother's education**			**Immunization **		
No education	64,297	31.3	NO/Partial	1,00,658	49.0
Primary	30,077	14.6	Full	1,04,822	51.0
Secondary	92,467	45.0	**Drinking water**		
Higher	18,639	9.1	Improved	1,79,628	87.4
**Caste**			Unimproved	25,852	12.6
Scheduled Caste	40,356	19.6	**Sanitation facility**		
Scheduled Tribe	43,267	21.1	Improved	1,02,259	49.8
OBC	83,373	40.6	Unimproved	1,03,221	50.2
Others	38,484	18.7			
Total			2,05,480		

*Composite Index of Anthropometric Failure

### Prevalence of anthropometric failure

Table 2 shows the prevalence of different forms of multiple anthropometric failures among the children by their background characteristics. It is found that a significant portion of the children (44%) in India do not show any kind of anthropometric failure but still a large proportion (56%) of the children carry some form of anthropometric failure and in many cases (34%) multiple anthropometric failures. It is found that the prevalence of multiple anthropometric failures among urban children (36%) is comparatively higher than the rural children (27%). The wealth pattern of CIAF shows that, children from the poorest wealth quintile show the highest prevalence of multiple anthropometric failures. It is observed that 9% of the poorest children are suffering from wasting & underweight, 10% of them suffer from wasting, stunting and underweight simultaneously while 27% of them suffer from stunting and underweight. The education (mother’s) pattern of anthropometric failure shows a decreasing prevalence among children with increasing educational attainment among their mothers. The prevalence of simultaneous occurrence of wasting, stunting and underweight is observed highest (9%) among those children whose mother did not achieve any formal educational qualification. Similarly, children of no educated mothers show highest prevalence (27%) in terms of concurrent occurrence of stunting & underweight and in terms of concurrent occurrence of wasting & underweight (9%). The social class pattern of CIAF shows that children from the scheduled caste and scheduled tribe category carry the higher burden of anthropometric failure than rest of the social groups. Birth order of the child shows differential in the prevalence of anthropometric failure. Higher ordered births show higher burden of multiple anthropometric failures. Around 66% of the children of the fourth or higher ordered births carry some form of the anthropometric failures. And it is observed that children of fourth or higher ordered births carry the highest (26%) prevalence of stunting and underweight. Male–female pattern of anthropometric failure does not show any substantial differential. Children aged between 2–5 years carry higher burden of CIAF than rest of the children. Similarly, the state pattern of anthropometric failures is shown in Table 3.

**Table 2:** Prevalence of anthropometric failure by background characteristics, India, 2015-16

Background Characteristics	No failure	Only wasting	Wasting and underweight	Wasting, stunting and underweight	Stunting and underweight	Only stunting	Only underweight	Total
**Place of residence**								
Rural	51.2	7.0	7.9	5.2	14.1	12.1	2.5	49,023
Urban	41.5	5.8	8.4	7.4	20.4	14.0	2.6	1,56,457
**Wealth quintile**								
Poorest	31.1	5.0	9.2	10.1	27.2	14.8	2.6	54,325
Poor	39.0	5.6	8.6	7.7	21.8	14.4	2.9	47,990
Middle	46.2	6.2	8.0	6.0	17.0	14.0	2.7	40,982
Richer	53.2	6.6	8.1	4.7	12.7	12.3	2.5	34,279
Richest	61.0	7.9	6.8	3.1	8.4	10.8	2.0	27,904
**Mother's education**								
No education	32.9	5.0	8.5	9.3	26.7	15.0	2.6	64,297
Primary	39.9	5.1	8.6	7.5	21.5	14.7	2.8	30,077
Secondary	49.2	6.7	8.4	5.6	14.8	12.8	2.6	92,467
Higher	62.1	8.4	6.5	2.9	7.6	10.4	2.1	18,639
**Caste**								
Scheduled Caste	40.5	5.7	8.2	7.4	21.0	14.6	2.7	40,356
Scheduled Tribe	35.8	6.3	10.6	10.1	21.6	12.7	3.1	43,267
OBC	44.3	6.1	8.0	6.5	18.8	13.8	2.6	83,373
Others	52.2	6.5	7.8	4.9	14.2	12.2	2.3	38,484
**Birth order**								
First	48.9	6.5	8.3	5.7	15.4	12.7	2.6	73,865
Second	45.5	6.3	8.2	6.4	17.6	13.4	2.7	63,748
Third	40.1	5.8	8.4	7.6	21.3	14.4	2.5	33,647
Fourth and more	33.8	4.9	8.2	9.4	26.4	14.9	2.4	34,220
**Sex of the child**								
Male	43.9	5.9	8.5	7.5	18.2	13.6	2.4	1,06,385
Female	44.5	6.3	8.0	5.9	19.1	13.4	2.8	99,095
**Place of delivery**								
Home	35.6	4.8	8.3	8.6	25.2	14.6	2.8	50,808
Institutional	46.5	6.5	8.2	6.3	16.9	13.2	2.5	1,54,672
**Child’s age**								
Less than 1 year	49.3	13.8	11.8	3.9	9.6	8.7	2.9	36,990
Between 1 and 2	41.7	5.3	8.3	8.6	16.9	17.6	1.7	41,147
Between 2 and 3	42.3	4.8	7.6	7.1	21.2	14.8	2.3	41,288
Between 3 and 4	43.2	4.1	6.8	7.1	22.3	14.0	2.5	43,795
Less than 5	45.0	3.7	7.5	6.7	21.7	11.8	3.5	42,260
**Immunization status**								
NO/Partial	42.9	7.5	8.6	6.5	19.0	12.9	2.6	1,00,658
Full	45.4	4.8	8.0	7.0	18.3	14.0	2.5	1,04,822
**Drinking water**								
Improved	44.0	6.1	8.2	6.7	18.8	13.6	2.6	1,79,628
Unimproved	45.6	6.3	8.7	7.1	17.3	12.5	2.6	25,852
**Sanitation facility**								
Improved	52.3	6.6	7.6	4.9	13.5	12.6	2.5	1,02,259
Unimproved	36.3	5.6	8.9	8.5	23.5	14.4	2.7	1,03,221
Total	44.2	6.1	8.3	6.8	18.6	13.5	2.6	2,05,480

**Table 3:** Prevalence of anthropometric failure by states, India, 2015–16.

States	No failure	Only wasting	Wasting & underweight	Wasting, stunting and underweight	Stunting & underweight	Only stunting	Only underweight	Total
Andaman And Nicobar Islands	57.3	10.1	6.0	3.3	10.3	10.9	2.1	537
Andhra Pradesh	52.5	4.7	7.1	5.4	17.1	10.0	3.2	2,243
Arunachal Pradesh	55.1	8.6	6.3	2.4	9.0	17.7	0.9	3,625
Assam	51.1	5.7	6.7	4.2	14.7	15.4	2.2	6,782
Bihar	36.1	5.0	7.6	8.3	25.8	14.6	2.5	21,165
Chandigarh	60.6	2.5	4.7	3.1	13.0	12.3	3.9	169
Chhattisgarh	42.0	6.6	10.5	6.3	17.8	13.5	3.4	7,714
Dadra & Nagar Haveli	35.3	9.6	11.7	6.5	19.7	15.9	1.3	271
Daman & Diu	53.7	12.0	9.2	3.4	12.4	7.7	1.5	323
Goa	59.8	8.3	7.1	2.2	10.7	8.6	3.3	315
Gujarat	40.3	7.9	10.5	8.2	18.6	12.1	2.6	5,965
Haryana	46.7	7.8	9.1	4.4	14.0	15.7	2.4	6,635
Himachal Pradesh	60.5	5.3	5.4	2.6	11.2	12.7	2.3	2,368
Jammu & Kashmir	57.9	5.9	4.8	2.6	11.5	16.4	1.0	4,377
Jharkhand	33.3	6.4	11.2	11.3	22.8	11.8	3.2	9,886
Karnataka	41.3	9.3	9.3	7.0	17.1	13.5	2.5	5,383
Kerala	64.5	8.3	6.0	1.8	7.2	10.8	1.5	2,028
Lakshadweep	60.6	4.1	6.4	3.7	11.3	11.6	2.4	278
Madhya Pradesh	37.7	6.8	10.2	8.8	21.2	12.4	3.0	19,794
Maharashtra	44.4	8.2	9.8	7.3	16.0	11.3	3.0	7,308
Manipur	64.7	3.3	2.1	1.4	9.2	18.3	1.1	4,959
Meghalaya	42.9	6.0	5.5	2.9	18.3	23.0	1.4	3,660
Mizoram	66.7	2.1	2.5	1.5	7.4	19.1	0.8	4,264
Nagaland	62.0	4.8	3.6	2.9	9.1	16.6	1.0	3,698
Delhi	55.3	5.8	4.9	4.7	16.3	11.2	1.7	1,113
Odisha	48.2	5.3	8.7	6.7	16.5	11.4	3.2	9,198
Puducherry	54.0	12.3	8.1	3.8	7.9	11.9	2.0	924
Punjab	60.4	5.3	6.6	3.7	9.6	12.7	1.7	4,611
Rajasthan	42.5	6.4	9.2	7.2	18.2	14.2	2.3	13,747
Sikkim	56.8	8.5	4.1	1.9	6.4	20.7	1.6	881
Tamil Nadu	55.4	7.9	7.7	4.2	10.1	12.9	1.8	6,598
Tripura	60.9	5.8	7.4	3.4	10.1	10.1	2.3	959
Uttar Pradesh	39.4	4.4	6.9	6.7	23.8	16.4	2.4	33,401
Uttarakhand	49.6	7.5	7.8	4.1	13.8	15.9	1.3	4,721
West Bengal	50.3	4.9	9.4	7.0	13.2	12.4	2.9	3,704
Telangana	56.7	6.0	5.8	5.8	14.0	8.7	3.0	1,876

### Risk factors of anthropometric failure in India, 2015–16

Four level random intercept model proposed in the methodology is applied to assess the socio-economic and demographic correlates of multiple anthropometric failures among Indian children within a multilevel framework (Table 4). In all three cases the statistically significant random intercept indicates considerable variation in concurrent occurrence of stunting & underweight, wasting & underweight and wasting, stunting & underweight among Indian children between states, between districts, between PSUs and among children.

**Table 4:** Multilevel logistic estimation of multiple anthropometric failures, India, 2015–16.

Background Characteristics	Stunting & underweight	Wasting & underweight	Wasting, stunting & underweight
**Place of residence**			
Rural^(Ref.)^	1	1	1
Urban	1.086**	1.076**	1.077**
**Wealth index**			
Poorest^(Ref.)^	1	1	1
Poorer	0.832**	0.859**	0.826**
Middle	0.665**	0.756**	0.691**
Richer	0.524**	0.688**	0.580**
Richest	0.387**	0.653**	0.458**
**Social class**			
SC^(Ref.)^	1	1	1
ST	0.944**	1.066*	1.057
OBC	0.893**	0.959*	0.918**
Others	0.767**	0.879**	0.767**
**Place of delivery**			
Home^(Ref.)^	1	1	1
Hospital	0.933**	0.942**	0.927*
**Mother’s education**			
No education^(Ref.)^	1		1
Primary	0.910**	0.950*	0.893**
Secondary	0.777**	0.903**	0.817**
Higher	0.524**	0.743**	0.609**
**Birth order**			
First^(Ref.)^	1	1	1
Second	1.068**	1.009	1.048*
Third	1.093**	1.029	1.081*
Fourth and more	1.165**	1.063*	1.156**
**Child's Age**			
Age	1.176**	0.943**	1.041**
**Child's sex**			
Male^(Ref.)^	1	1	1
Female	0.933**	0.823**	0.729**
**Immunisation status**			
No^(Ref.)^	1	1	1
Partial	1.003	1.002	1.009
Full	1.144**	1.065*	1.215**
**Sanitation**			
Improved^(Ref.)^	1	1	1
Unimproved	0.942**	0.992	0.969
**Drinking water**			
Improved^(Ref.)^	1	1	1
Unimproved	1.064**	1.065**	1.075*
**Random effect**			
Ωf	0.128**	0.134**	0.193**
Ωv	0.057**	0.122**	0.130**
Ωu	0.212**	0.298**	0.566**
State (f)	3.47%	3.50%	4.64%
District (v)	1.55%	3.19%	3.13%
PSU (u)	5.75%	7.79%	13.61%
Child (error)	89.23%	85.51%	78.62%

**p value < 0.01; *p-value < 0.05; ^(Ref.)^ Denotes the reference group.

The concurrent occurrence of stunting & underweight is 1.09 (AOR = 1.086; p-value < 0.01) times more likely among the children residing in urban areas than rural areas (Table 4). The odd is almost same for the other two categories of multiple anthropometric failures (AF) among the urban children. The economic status of a household is inversely associated with each of the indicators of multiple AF. Children from the richest wealth quintile are 61% less likely (AOR = 0.39; p-value < 0.01) to suffer from stunting & underweight, 35% (AOR = 0.65; p-value < 0.01) less likely to suffer from wasting & underweight and 54% (AOR = 0.46; p-value < 0.01) less likely to suffer from the simultaneous occurrence of wasting, stunting and underweight than those children from the poorest wealth quintile. Similarly, children from the middle and richer wealth quintiles are also substantially less likely to suffer from multiple AF. Among the children from different social groups; ST, OBC and Others show comparatively lower likelihood to multiple AF than the reference category which means children from SC category bear the highest risk of stunting & underweight, wasting & underweight and wasting, stunting & underweight. Mother’s educational attainment shows a very strong statistical association with each of the indicators of multiple AF. Thus it is observed that children of higher educated mother are 48% (AOR = 0.52; p-value < 0.01) less likely to suffer from stunting & underweight, 26% (AOR = 0.74; p-value < 0.01) less likely to suffer from wasting & underweight and 39% (AOR = 0.61; p-value < 0.01) less likely to suffer from wasting, stunting and underweight than those children of no educated mothers. Children of secondary and primary educated mother are also showing lower odds to suffer from each of the types of multiple AFs. Children of higher birth orders show comparatively higher odds of multiple AF. Children of four or higher ordered birth show 17% (AOR = 1.17; p-value < 0.01) higher chance of stunting & underweight and 16% (AOR = 1.16; p-value < 0.01) higher chance of wasting, stunting and underweight than the first ordered births. Apparently, female children show lower likelihood to multiple AF than the male children. It is also found that fully immunized children carry higher likelihood to multiple AF than the rest of the children. Like, fully immunized children are 14% (AOR = 1.14; p-value < 0.01) more likely to be stunted & underweight and 22% more likely to be wasted, stunted & underweight. .

### Geographical variation in multiple anthropometric failures

The estimated inter-state variance is observed highest (Ω_f_ = 0.19; p-value < 0.01) for simultaneous occurrence of wasting, stunting & underweight followed by concurrent occurrence of wasting & underweight (Ω_f_ = 0.13; p-value < 0.01) and stunting & underweight (Ω_f_ = 0.13; p-value < 0.01). The corresponding variance partition coefficient (VPC) value shows that 3.5% of the overall variation in the concurrent occurrence of stunting & underweight among children is due to systematic variation in the states while the corresponding values are 3.5% and 4.6% for wasting-underweight and wasting, stunting & underweight respectively. Similarly, district, PSU and children level systematic variations are measured for each of the indicators of AFs and are shown in Table 4. It is found that, the PSU level systematic variation is observed highest (14%) for wasting, stunting & underweight than rest of the two multiple anthropometric failures.

### District level patterns of infant and child mortality

Table 5 shows the estimates of average infant and under-five mortality by district level characteristics. Districts are characterised in terms of the concurrent occurrence of stunting & underweight, concurrent occurrence of wasting & underweight, concurrent occurrence of wasting, stunting & underweight and other socio-economic and demographic factors. Among all other characteristics, district level burden of multiple anthropometric failures are considered to be the key exposure variables for infant and child mortality. The prevalence of stunting & underweight varies between 2–36% across the districts and it is observed that districts with lower prevalence of stunting & underweight show lower prevalence of infant and child mortality (per 1000 live births). It is estimated that districts with (23–36) % prevalence of stunting & underweight show an average infant mortality rate of 53 per 1000 live births and average CMR of 14 per 1000 live births. In the same direction, it is found that districts with higher prevalence in terms of wasting & underweight show higher burden of infant and child mortality. Districts where the prevalence of concurrent occurrence of wasting & underweight ranges in between (14–23)% show an average IMR of 41 and CMR of 11 per 1000 live births. Similarly, higher prevalence of wasting, stunting and underweight across the districts show higher burden of IMR and CMR across the districts. Among the other socio-economic and demographic characteristics of the districts, proportion of rural, proportion of poor, proportion of no educated mothers, proportion of higher ordered births, percentage of full immunization and proportion of children with no access to improved sanitation show substantial differential in IMR and CMR across the districts.

**Table 5:** Average infant & child mortality per 1000 live births by district level characteristics, India, 2015–16.

District level characteristics (%)	Infant mortality*	Child mortality*
**Concurrent prevalence of stunting & underweight**		
2–13	26	5
13–23	40	9
23–36	53	14
**Concurrent prevalence of wasting & underweight**		
0–7	37	8
7–14	39	9
14–23	41	11
**Concurrent prevalence of wasting, stunting & underweight**		
0–7	35	8
7–14	44	10
14–21	43	14
**Proportion of rural population**		
0–20	43	10
20–60	34	7
> 60	26	6
**Proportion of poor**		
0–30	26	5
30–60	39	8
> 60	49	12
**Proportion of no educated mothers**		
0–30	31	6
30–60	48	12
60–93	56	16
**Proportion of SC/ST**		
< 30	39	8
30–60	38	8
> 60	35	11
**Proportion of four or higher ordered births**		
< 15	31	6
15–30	48	12
30–42	55	14
**Proportion of female child**		
35–50	40	9
50–63	34	9
**Percentage of home delivery**		
0–30	35	8
30–60	48	12
60–90	35	12
**Percentage of no/partial immunization**		
< 40	31	5
40–70	41	10
70–95	42	13
**Proportion of children with no access to safe drinking water**		
< 30	38	8
30–50	41	12
50–69	31	8
**Proportion of children with no access to improved sanitation**		
< 30	28	5
30–60	35	8
60–93	49	12

*Per 1000 live births.

**Figure 1:**
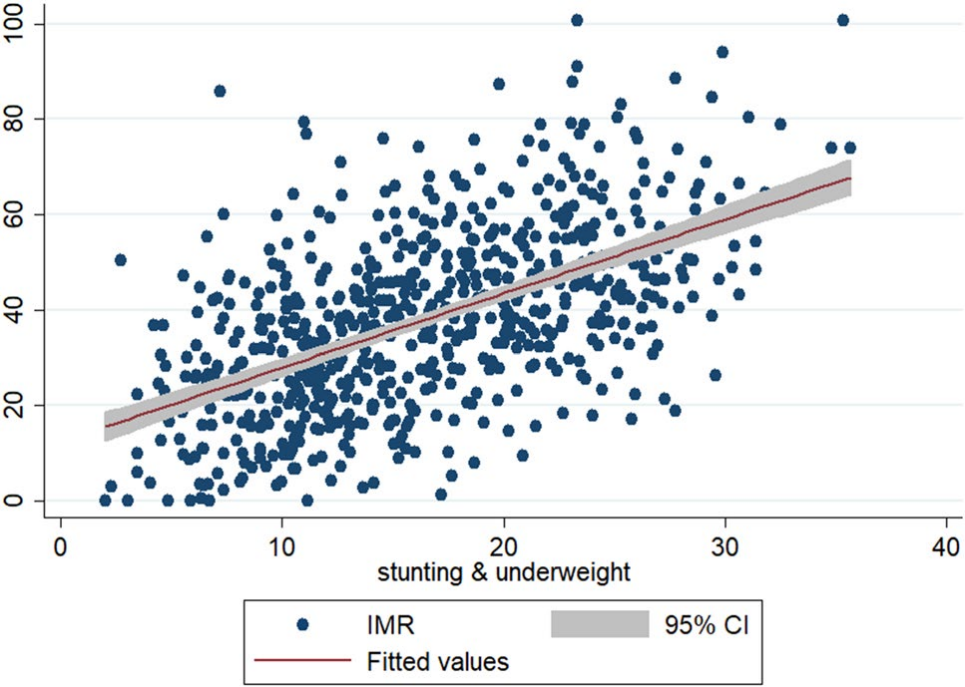
Scatter plot between district level infant mortality rate (IMR) and the prevalence of concurrent occurrence of wasting & underweight, India, 2015–16.

**Figure 2:**
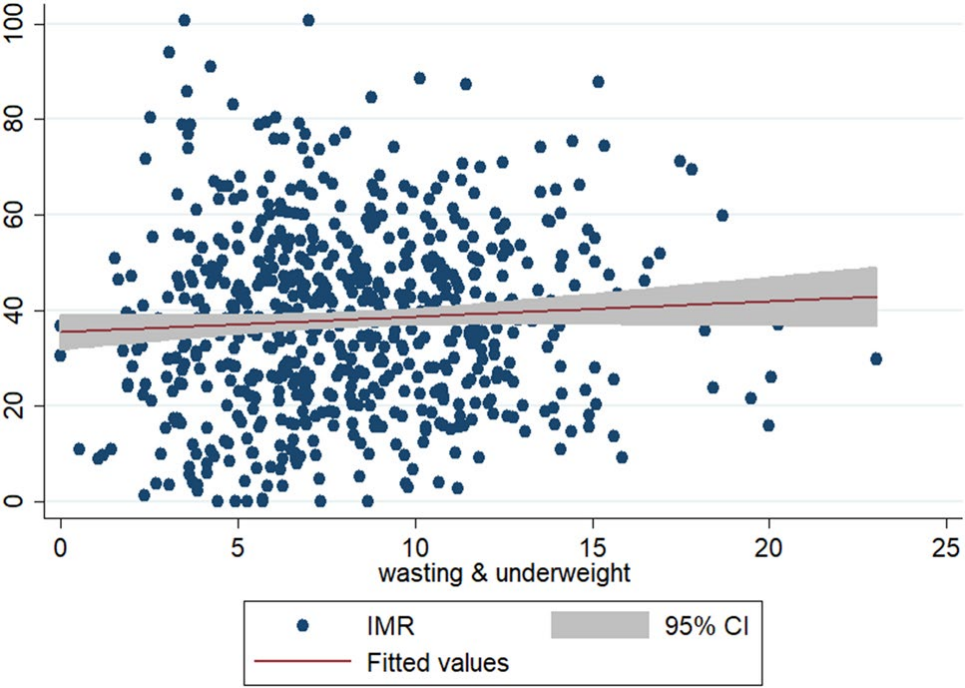
Scatter plot between district level infant mortality rates (IMR) and the prevalence of concurrent occurrence of wasting & underweight, India, 2015–16.

**Figure 3:**
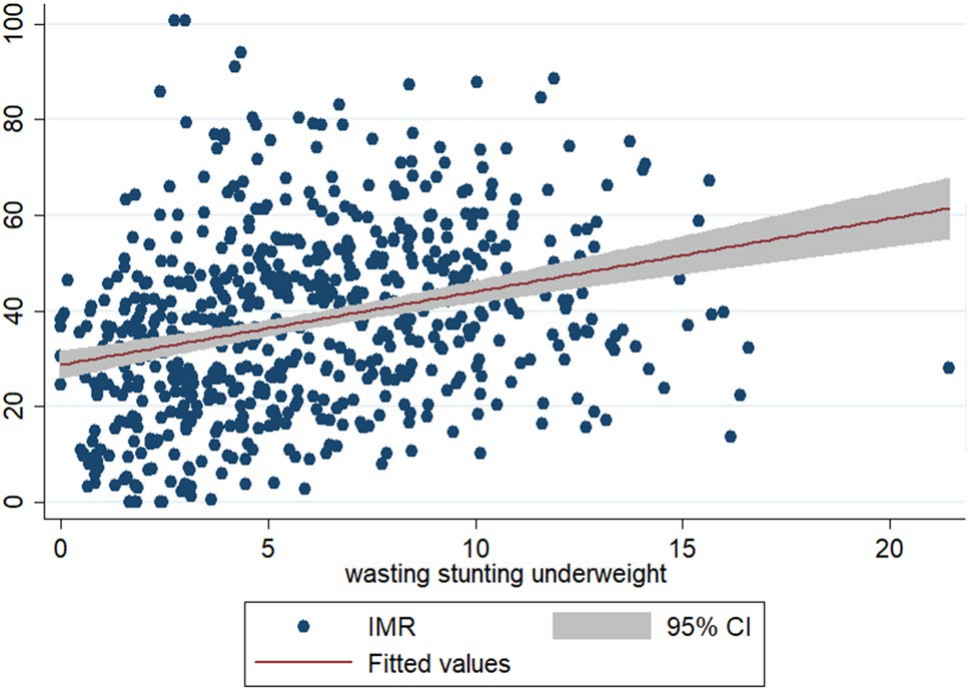
Scatter plot between district level infant mortality rates (IMR) and the prevalence of concurrent occurrence of wasting, stunting & underweight, India, 2015–16.

**Figure 4:**
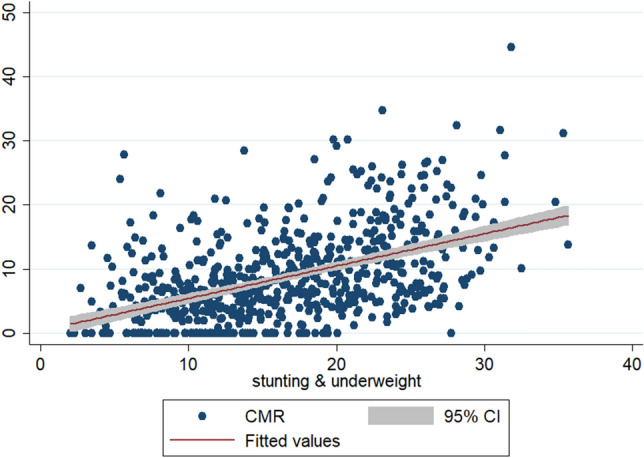
Scatter plot between district level child mortality rates (CMR) and the prevalence of concurrent occurrence of stunting & underweight, India, 2015–16.

**Figure 5:**
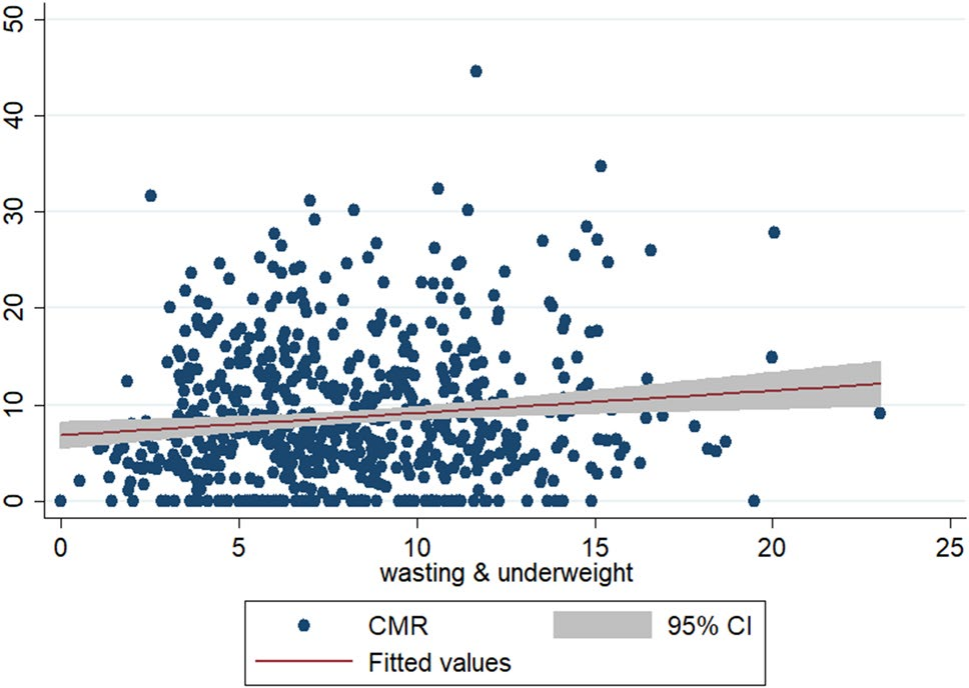
Scatter plot between district level child mortality rates (CMR) and the prevalence of concurrent occurrence of stunting & underweight, India, 2015–16.

**Figure 6:**
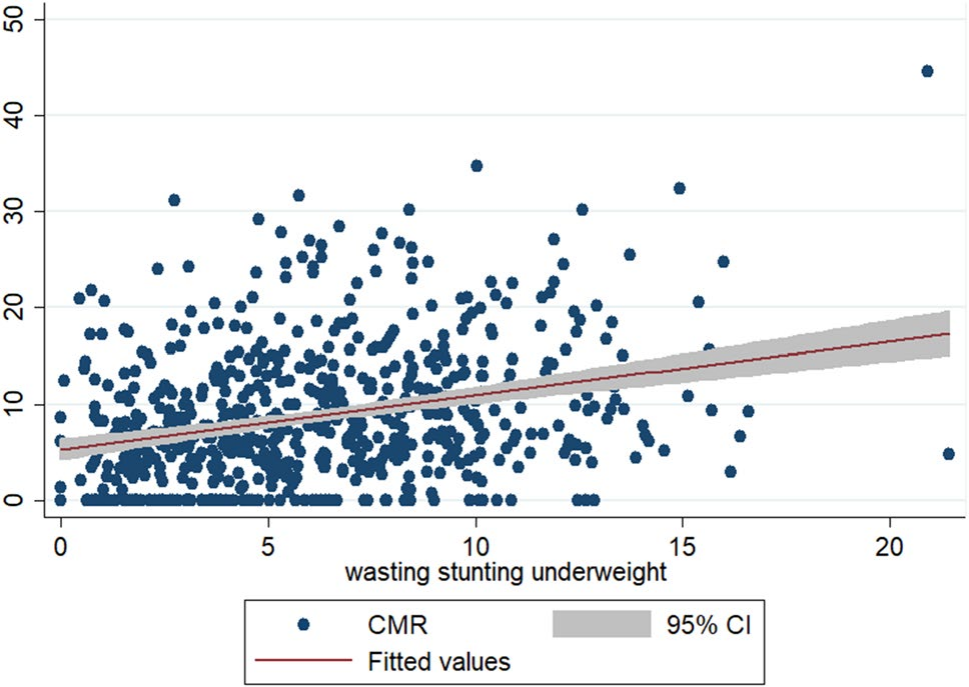
Scatter plot between district level child mortality rates (CMR) and the prevalence of concurrent occurrence of wasting, stunting & underweight, India, 2015–16.

To check the scatteredness of district level rates of infant and child mortality we plotted the two way scatter plots between the measures of mortality and the measures of multiple anthropometric failures. Figures 1, 2, 3 show the scatter plots of IMR and Figs. 4, 5, 6 show the scatter plots of CMR. From the figures it is evident that the points are not exactly clustered along the line of mean. Though it is observed that higher burden of stunting & underweight and wasting, stunting & underweight across the districts of India show higher burden of IMR and CMR across the districts.

### District level risk factors of infant mortality

Extending the traditional regression models, the application of generalised linear model allows us to model the district level mean response of IMR based upon the explanatory variables under the study framework through the log link function which assumes the response variable to be a member of exponential family. As both the *Shapiro–Wilk* (W = 0.989; p-value = 0.0001) and the *Shapiro-Francia* (W = 0.992; p-value = 0.0017) normality test confirmed the district level distribution of IMR to be non-normal and we assumed the outcome of interest to be a member of the exponential family because the response variable is discrete and positively skewed. And at the same time, the exponential family gives a better model fit than rest of the assumptions. The generalised linear model with exponential family and log link assumes the multiplicative effects on the original outcome by the predictors.

Model 1a gives the estimated coefficients for stunting & underweight (Table 6). Here the coefficients show the magnitude of increase or decrease in the log arithmetic mean of IMR. It is found that district level prevalence of concurrent occurrence of stunting & underweight significantly predicts the district level burden of infant mortality across India. The estimated coefficient (β) of “stunting & underweight” is 0.017 (p-value < 0.001)) which indicates a strong association between concurrent occurrence of stunting & underweight among the children and infant mortality across the districts of India. This suggests that the probability of infant mortality among the children under age one increases with the increase in the prevalence of concurrent occurrence of stunting & underweight among the children across the districts. It is evident that one unit increase in the prevalence of concurrent occurrence of stunting & underweight is statistically associated with 0.017 unit increase in the log arithmetic mean of IMR when adjusted for other variables. More specifically, the log arithmetic mean of IMR will be 1.017 times higher with every one unit increase in the rate of concurrent occurrence of stunting & underweight across the districts. Model 1b also shows a statistically significant association (β = 0.005; p-value = 0.008) between the concurrent occurrence of wasting & underweight and IMR. This indicates the districts with higher concurrent occurrence of wasting & underweight bear higher infant mortality. Similarly, the district level prevalence of concurrent occurrence of wasting, stunting & underweight does appear to be highly statistically significant predictor of district level IMR (Model 1c). From the model estimation, it is observed that district level variation in terms of each of the multiple anthropometric failures significantly predicts the district level variation in infant mortality in India.

**Table 6:** Generalised Linear Model estimation of IMR, India, 2015–16.

Model 1a			Model 1b			Model 1c		
Variables (%)	Coef. (95% CI)	P > z	Variables (%)	Coef. (95% CI)	P > z	Variables (%)	Coef. (95% CI)	P > z
Stunting and underweight	0.017(0.014–0.020)	0.000	Wasting & underweight	0.005(0.001–0.009)	0.008	Wasting, stunting & underweight	0.010(0.006–0.014)	0.000
Rural	-0.001(-0.002–0.000)	0.005	Rural	-0.001(-0.002–0.000)	0.037	Rural	-0.001(-0.002–0.000)	0.016
Poor	0.003(0.002–0.004)	0.000	Poor	0.004(0.003–0.005)	0.000	Poor	0.004(0.003–0.005)	0.000
No education	0.000(-0.002–0.001)	0.469	No education	0.000(-0.001–0.002)	0.524	No education	0.000(-0.001–0.001)	0.942
SC/ST	-0.003(-0.003–0.002)	0.000	SC/ST	-0.004(-0.004–0.003)	0.000	SC/ST	-0.004(-0.004–0.003)	0.000
Four plus ordered births	0.014(0.012–0.016)	0.000	Four plus ordered births	0.018(0.016–0.020)	0.000	Four plus ordered births	0.018(0.016–0.020)	0.000
Female child*	-0.001(-0.002–0.001)	0.455	Female child*	0.000(-0.002–0.001)	0.958	Female child*	0.000(-0.002–0.001)	0.932
Home delivery*	0.000(-0.001–0.001)	0.825	Home delivery*	0.000(-0.001–0.001)	0.628	Home delivery*	0.000(-0.001–0.001)	0.755
No/partial immunization*	0.001(-0.001–0.003)	0.278	No/Partial immunization*	0.001(-0.001–0.003)	0.263	No/Partial immunization*	0.001(0.000–0.003)	0.130
Unimproved drinking water	0.027(0.000–0.054)	0.052	Unimproved drinking water	0.074(0.049–0.100)	0.000	Unimproved drinking water	0.066(0.040–0.091)	0.000
Unimproved sanitation	-0.014(-0.047–0.020)	0.416	Unimproved sanitation	-0.036(-0.070–0.003)	0.031	Unimproved sanitation	-0.036(-0.069–0.003)	0.034
AIC	11.98	AIC	12.16	AIC	12.13			

Model 1a: IMR = f (stunting & underweight, control variables).

Model 1b: IMR = f (wasting & underweight, control variables).

Model 1c: IMR = f (wasting, stunting & underweight, control variables).

*Proportion calculated out of children aged between [0–1) year.

Notably, variables like district level proportion of poor, proportion of four or higher ordered births, district level proportion of households with unimproved drinking water (except Model 1a) and unimproved sanitation show statistically significant and positive association with IMR throughout the models. This indicates that district level increase in poverty (proportion of poor) is associated with increase in (β = 0.003; p-value < 0.001) IMR among children (Model 1a). Similarly, districts with higher percentages of four or higher ordered births carry higher burden of IMR. This suggests that infant mortality is much more prevalent among the children of higher ordered births. Drinking water and sanitation situation across the districts also predict the variation in infant mortality. The likelihood ratio test statistic found statistically significant which suggests that comparability between the full and reduced models providing the evidence of non-null parameter estimates against the null hypothesis, H_0_: β_1_ = β_2_ = …β_n_ = 0.

### District level risk factors of child mortality

Like IMR, we modeled child mortality within the same study framework and we found that prevalence of each of the forms of multiple anthropometric failures is significantly associated with CMR burden across India (Table 7). Model 2a shows an estimated coefficient for stunting & underweight to be 0.029 (p-value < 0.001). Similar to Model 1a, Model 2a also carries statistical evidence on anthropometric failure (stunting & underweight) and child mortality burden across the districts of India. Like, concurrent occurrence of stunting & underweight among children, concurrent occurrence of wasting & underweight and simultaneous occurrence of wasting, stunting & underweight also closely predicts the district level burden of CMR in India. This shows that districts with higher burden of multiple anthropometric failures are at more risk of higher child mortality burden. Unlike IMR, the district level prediction of CMR shows that mother’s educational status and immunization status of the children substantially predict the district level variation in CMR. The estimated beta coefficients also suggest poverty association of district level CMR burden. The CMR framework shows lower AIC values for each of the forms of anthropometric failures than the IMR. Similar to IMR model estimation, the likelihood ratio test for CMR provides the evidence of non-null parameter estimates against the null hypothesis, H_0_: β_1_ = β_2_ = …β_n_ = 0 from the GLM estimation.

**Table 7:** Generalised linear model estimation of CMR, India, 2015–16.

Model 2a			Model 2b			Model 2c		
Variables (%)	Coef. (95% CI)	P > z	Variables (%)	Coef. (95% CI)	P > z	Variables (%)	Coef. (95% CI)	P > z
Stunting and underweight	0.029(0.023–0.035)	0.000	Wasting & underweight	0.022(0.014–0.030)	0.000	Wasting, stunting & underweight	0.020(0.012–0.029)	0.000
Rural	0.003(0.001–0.005)	0.003	Rural	0.003(0.001–0.005)	0.002	Rural	0.003(0.001–0.005)	0.002
Poor	0.002(0.000–0.004)	0.066	Poor	0.002(0.000–0.005)	0.034	Poor	0.003(0.000–0.005)	0.029
No education	0.004(0.002–0.006)	0.001	No education	0.005(0.002–0.007)	0.000	No education	0.004(0.002–0.007)	0.001
SC/ST	0.004(0.003–0.005)	0.000	SC/ST	0.002(0.000–0.003)	0.011	SC/ST	0.002(0.001–0.003)	0.001
Four plus ordered births	0.015(0.010–0.019)	0.000	Four plus ordered births	0.023(0.019–0.028)	0.000	Four plus ordered births	0.022(0.017–0.027)	0.000
Female child*	0.001(-0.006–0.008)	0.780	Female child*	0.001(-0.006–0.008)	0.726	Female child*	0.000(-0.007–0.008)	0.891
Home delivery*	0.001(-0.001–0.003)	0.293	Home delivery*	0.001(0.000–0.003)	0.155	Home delivery*	0.001(-0.001–0.003)	0.182
No/partial immunization*	0.004(0.002–0.006)	0.000	No/partial immunization*	0.005(0.003–0.007)	0.000	No/partial immunization*	0.005(0.003–0.007)	0.000
Unimproved drinking water	0.099(0.039–0.158)	0.001	Unimproved drinking water	0.171(0.115–0.227)	0.000	Unimproved drinking water	0.162(0.105–0.219)	0.000
Unimproved sanitation	0.097(0.030–0.164)	0.004	Unimproved sanitation	0.052(-0.015–0.118)	0.126	Unimproved sanitation	0.057(-0.008–0.123)	0.088
AIC	7.50	AIC	7.59	AIC	7.60			

Model 2a: CMR = f (wasting & underweight, control variables).

Model 2b: CMR = f (wasting & underweight, control variables).

Model 2c: CMR = f (wasting, stunting & underweight, control variables).

*Proportion calculated out of children aged between [1–4] year.

## Discussion

Almost two-fifth of all the children in India suffers from chronic undernutrition and still infant and under five mortality is substantially high in India^12^. Though there are different measures of undernutrition; still, due to some demerits of the given scientific measure, we may fail to identify the group of children with multiple anthropometric failures. In this context, this study examined the pattern and severity of multiple anthropometric failures among under-five children by population characteristics and across states of India. Additionally, the district level burden of infant and child mortality is predicted in terms of the different forms of anthropometric failure prevailing across India. Among the three anthropometric measures of undernutrition- stunting is defined as the chronic and long term undernutrition and wasting being the acute form of undernutrition, while the measure of underweight is assumed to be a combination of stunting and wasting both and indicates a possible occurrence of short and long term of undernutrition^37^. Thus in a group of children undernourished, the possible anthropometric failures could be multiple and severe which needs a careful identification of the exact type of growth faltering among the under-five children. In this context, this study provides a detailed understanding of the current scenario on multiple anthropometric failures prevailing among under-five children in India using the most recent household based survey data available for India. Thus we examined the risk factors of multiple anthropometric failures within a multi-level framework taking care of the hierarchy of the survey dataset. Additionally, we performed a district level analysis to predict the district level burden of infant and child mortality in terms of the prevalence of multiple anthropometric failures.

A significant proportion of the children across India carry different forms of anthropometric failures and concurrent occurrence of “stunting and underweight” is much more prevalent followed by “wasting & underweight” and “wasting, stunting & underweight”. Of the different types of anthropometric failures, the prevalence of concurrent occurrence of “stunting & underweight” shows high national average with sharp differential by place of residence (rural/urban), wealth quintile, mother’s education, birth order, place of delivery and child age. Notably, no substantial gender difference in terms of any of the identified anthropometric failures is observed. Parallel to the concurrent occurrence of “stunting & underweight” it is also observed that the prevalence of “only stunting” is also quite high by different socio-economic characteristics of the children. It is observed that the burden of “only underweight” shows the lowest prevalence than any other forms of anthropometric failures. The state level pattern of CIAF among under-five children shows significant variation. And the largest variation is observed in terms of “stunting & underweight”. More than one-fifth of the total children from the states like Bihar, Uttar Pradesh, Jharkhand and Madhya Pradesh are both stunted and underweight. And at the same time it is also alarming to note that 8–11% of the total children from the states like Jharkhand, Madhya Pradesh, Bihar and in Gujarat suffer from stunting, underweight and wasting simultaneously.

Among Indian children, the risks of different forms of multiple anthropometric failures are significantly determined by their socio-economic and demographic characteristics. Within the multilevel framework, we find the hierarchy in the variations of multiple anthropometric failures. It is found that children from the urban areas carry higher odds to suffer from multiple anthropometric failures-stunting & underweight, wasting & underweight and wasting and stunting and underweight. The wealth pattern of multiple anthropometric failures show a gradual reduction in odds across the richer wealth quintiles and children with higher economic wellbeing show lower odds in terms of all the three types of anthropometric failures. This wealth gradient of anthropometric failure clearly suggests a very sharp differential in the burden of anthropometric failures and children from the lower wealth quintiles are extremely vulnerable to multiple anthropometric failures. Similar to wealth gradient, the education (mother’s) gradient is also evident. From the estimated AOR values it is clear that higher educational attainment among the mothers significantly reduces the likelihood to suffer from multiple anthropometric failures among their children-especially for the concurrent occurrence of stunting & underweight and concurrent occurrence of wasting, stunting and underweight. Apparently, higher ordered births show higher likelihood to anthropometric failure. The odds of multiple anthropometric failures show persistent increase among the second, third and four or higher ordered births compared the first ordered births. Previous studies also suggest poor nutritional health among children of higher ordered births subject to food insecurity of the households^38, 39^. It is argued that, households with low or no food security may substantially face food shortage causing the children to starve and thus malnourished^40–42^. Though observed gender pattern of anthropometric failure does not show any differential but once adjusted for other covariates, it is found that female children are comparatively less likely to suffer from multiple anthropometric failures. Though in Indian setting, it is profoundly argued on the anti-female bias of food allocation^43^ across the households of different socio-economic and demographic characteristics but the present study carries the evidence of lopsided low likelihood to anthropometric failure among female children than the males which is a consistent finding at par with the previous studies^44^. This study also finds that children with no access to improved source of drinking water show higher likelihood to different forms of anthropometric failures, consistent with the findings of previous studies showing the linkages between child malnutrition and drinking water^45, 46^.

The district level aggregated analysis of the data brings forth the pattern of infant and child mortality in terms of the prevailing district level burden of multiple anthropometric failures among the children under age five. The bivariate analysis clearly demonstrates higher infant and child mortality in those districts where the prevalence of anthropometric failure is comparatively higher. GLM estimation of the district level rates of infant and child mortality indicates that districts with higher prevalence of multiple anthropometric failures show elevated risk of infant and child mortality. Each of the forms of multiple anthropometric failures shows strong statistical association to explain the district level variation in IMR and CMR. Previous literatures suggest that poor nutrition is associated with immuno-deficiency against the infectious diseases among children and undernourished children are at higher risk of dying from diseases like malaria, measles, diarrhea and acute respiratory infections^7, 47, 48^. Low plasma complement, reduction in exocrine secretion of protective substances, poor gut function and reduction in antibodies produced after vaccination are common among malnourished children who are susceptible to higher risk of death. Although, cause-specific mortality is not known but this study confirmed that districts with higher prevalence of multiple anthropometric failures are at higher risk of experiencing increased infant and child mortality.

Apart from the children’s nutritional status, other district level characteristics like- poverty situation across districts shows substantial association with IMR. It is also evident that IMR is much more prevalent among the children of higher ordered births across the districts of India. Availability of clean and safe drinking water also shows an interplay with IMR across the districts and districts with higher portion of children with no access to improved source of drinking water show increased risk of IMR. In case of CMR, other than poverty, mother’s education, children’s immunization status, drinking water and sanitation condition show substantial association with CMR. At the aggregated level, the gender pattern of infant and child mortality shows no statistical significance. Although, district level proportion of rural shows negative association with IMR; while, the association with CMR has been found positive and suggests that among the rural children the risk of CMR is higher. Contrary to the CMR situation, the risk of infant mortality is found lower among the rural children than the urban children when adjusted for other district level risk factors.

The study has few limitations. First, this study is based upon a cross-sectional study whereby we cannot draw any causal inference. Second, though within a cross-sectional framework we checked the pattern of CIAF prevalence by population characteristics and examined the determinants yet the data information was limited. Like, child’s nutritional status directly depends upon their previous disease pattern however; there is no appropriate information available on this aspect of the children from the dataset. Third, this study used the last five years birth history and included all the children surveyed but for a significant portion of the sample, we have either the anthropometry data missing or the children’s height/weight measure are out of the plausible limits and thus we had to drop a number of 54,147 many children from the data set. Additionally, checking the pattern of missing we found that the missing pattern is not random (MAR) and thus we could not impute. Though we followed the DHS guidelines, another limitation of vaccination data from National family Health Survey is that it is based upon mother’s recall when vaccination card not found at the time survey, which introduces a non-sampling bias in the estimation process^50, 51^. Additionally, the dataset lacks the information on cause specific mortality and in a country like India, diarrhoeal infection, acute respiratory infection and low utilization of post natal care largely contributes to child mortality^37, 52, 53^ with state and regional variation to which we could not adjust while compiling the infant and child mortality rates to execute the district level meso scale analysis.

## Conclusion

This study assessed the multilevel contextual determinants of anthropometric failures among Indian children and further explored the district level variation in IMR and CMR in terms of the existing burden of multiple anthropometric failures. This study shows that a significant portion of children under age five in India suffers from multiple anthropometric failures. And socio-economic and demographic characters of the children show larger variation in different types of anthropometric failures-especially in terms of the concurrent occurrence of stunting & underweight in India. Analysis suggests that household’s economic wellbeing and mother’s education plays a significant role in child’s nutritional status. The multilevel analysis of CIAF confirms significant geographical variations in terms of each of the anthropometric failures across districts, across PSUs within districts and among the children within PSUs. The children level variation in multiple anthropometric failures is found to be the largest. This reiterates the fact that anthropometric failure among children in India is a micro-level phenomenon than a subject at the meso scale. At the child level, mother’s education, birth order of the child and sex of the child substantially determine the different forms of anthropometric failures. At the same time, household’s economic status in terms of wealth plays a crucial role on child’s nutrition. Although it has shown a reduction over time, Indian districts are still burdened with high levels of IMR and CMR and this study propagates the key message that districts with higher burden of multiple anthropometric failures are vulnerable and exposed to higher burden of infant and child mortality in India.

## Supplementary information

Supplementary Information 1.
