# Supplementary material for: The burden of anthropometric failure and child mortality in India
Source: Sci Rep. 2020 Dec 2;10:20991. doi: 10.1038/s41598-020-76884-8 (PMC7710716; doi:10.1038/s41598-020-76884-8)
Supplement: Supplementary file 1 — Supplementary Information 1. [file 41598_2020_76884_MOESM1_ESM.docx]

| Title | **The Burden of Anthropometric Failure and Child Mortality in India** |
| --- | --- |
| Author’s Name | Junaid Khan^1*^ & Sumit Kumar Das^2^ |
| Authors’ Affiliation | ^1^ Doctoral Fellow,  International Institute for Population Sciences,  Govandi Station Road, Mumbai-400088,  Maharashtra, India  E-mail address: statjun@gmail.com  ^2^Doctoral Fellow,  Dept. of Biostatistics,  National Institute of Mental Health and Neurosciences ,  Bengaluru-29, India  E-mail address: [sumitdas382@gmail.com](mailto:sumitdas382@gmail.com) |
| ^*^Corresponding Author | Doctoral Fellow,  International Institute for Population Sciences,  Govandi Station Road, Mumbai-400088,  Maharashtra, India  E-mail address: statjun@gmail.com  Mobile Contact: +91-9653601397 |

**Annexure-1**

**Flow chart showing the analytical sample**

#Children for whom the social caste information is missing=8,429

#Children not being a de jure resident and the type of toilet facility is unknown=11,374

#Children not being a de jure resident and the source of drinking water is unknown=11,374

Final analytical sample=2,05,480

Missing cases=23,172

Flagged cases=10,235

#Children with age in days out of plausible limits=1,197

#Children with height out of plausible limits=1,197

Children for whom, the anthropometric z-score is available=2,25,002

Total No of Children surveyed aged 0-59 months=259,627
